# Pilot simulation-based research for evaluation, training and assessment of holistic nursing at four intensive care unit sites

**DOI:** 10.15694/mep.2019.000040.1

**Published:** 2019-03-07

**Authors:** Tsu-Hui Shiao, Chen-Yi Wu, Ying-Ying Yang, Shi-Chuan Chang, Boaz Shulruf, Ling-Yu Yang, Chen-Huan Chen, Fa-Yauh Lee

**Affiliations:** 1 High-fidelity Medical Simulation Center for Holistic Care and Inter-Professional Collaboration; 2 Department of Chest Medicine; 3 New South Wales Syndney University; 4 National Yang-Ming University

**Keywords:** holistic care, teamwork, intensive care unit, workplace assessment

## Abstract

This article was migrated. The article was marked as recommended.

**Background:** Holistic nursing of intensive care unit (ICU) patients encompasses diverse challenges requiring regular in situ evaluation, training and assessment. Simulation has been adopted as a research and training tool in medicine; however, evidence for its use in enhancing holistic care at multi-sites is limited.

**Objective:** This study aims to conduct a simulation-based research (SBR) at four ICU for standardized training of holistic nursing.

**Methods:** There are stages of evaluating, training+in-training assessment and post-course assessment in this SBR. Specifically, the curriculum-mapped scenarios were developed according to the deficiency of each site after evaluating stage. At the training stage, the first simulation by team was defined as preparation step and the in-training assessment was undertaken at the second simulation.

**Results:** From January 2017 to October 2018, sixty-four ICU nurses (16 teams, 4 teams in each site) at RCU, PICU, NICU and GYN ICU, attend 8 similar courses (2 courses at each site) over 20 months. In comparison with baseline performance, in-training assessments revealed the significant improvement of attendee’s skills of holistic nursing. Attendees commented that simulation was a valuable training modality to enhance skills of holistic care including history taking, physical examination, communication and teamwork that are rarely taught among ICU nurses. Post-course workplace assessment by senior nurses revealed the high frequency of clinical application of holistic nursing by attendees. Additionally, post-course self assessment revealed a high attendee’s confidence of holistic approaching in ICU. Conclusion: This pilot SBR demonstrated the feasibility of a standardized holistic care simulation program across four ICUs. In situ simulation and post-course workplace assessment affords situational learning without compromising patient safety and is an exciting and novel training of holistic nursing for ICU that could be integrated into regular intervention.

## Introduction

Healthcare professionals training in holistic care are required to develop a variety clinical skill to facilitate their management of a complex and challenging patient population. Facing the heightened needs of critical patients, intensive care unit (ICU) nurses need to provide holistic care to patients and families by assessing patients, and integration of the opinion of healthcare team by communication and collaboration ( Pelletier, 2014; Steaban, 2016; Crouch, 2016). In ICU, nurses are being most accessible to the patient and families. So, ICU nurses are expected to be highly experienced and knowledgeable healthcare professionals that familiar with holistic nursing.

It had been reported that teaching holistic nursing to nursing students is ignored in the curriculum ( King, 2006). Meanwhile, defective teaching methods and educators’ incompetence are barriers to training holistic nursing on campus ( Henderson, 2002). Compatibility between personality and profession as well as nurses’ knowledge are crucial for the success for the provision of holistic care ( King, 2006; McEvoy, 2008). So, some nurses are not familiar with holistic care due to improper professional relations and limited knowledge ( Selimen, 2011; Keegan, 1987).

Simulation-based nursing education is an increasingly popular pedagogical approach to train novice as well as experienced nurses. It provides attendees with opportunities to practice their technical and non-technical skills through rare authentic life-threatening real-world situational experiences ( Kim, 2016). Additionally, simulation had been suggested as a research tool to evaluate the effects of special educational intervention ( Cheng, 2014). Post-simulation workplace assessment can assess the transference of learnt skills and knowledge of attendees on routine practice.

Skills of history taking, physical examination, effective communication and teamwork are essential for holistic care on psychological, social, and spiritual dimensions. However, these skills are traditionally associated with doctors rather than essential skills of nurses ( Marsden, 2003). Moreover, technological advances and working hour restriction may lead physicians omit their aforementioned roles in ICU. Especially, the physicians-in-the-lead strategy appears to be insufficient for holistic healthcare delivery ( Malik, 2018). Clinically, nurses are often the first responder to examine and handle the acute deterioration of ICU patients ( Guillamet, 2015). Actually, lack of continuous workplace evaluation/training and lack of support from team member across other professions such as physicians are significant main barrier for experienced ICU nurses to do holistic nursing ( Osborne, 2015).

Taking together, our study uses workplace inter-professional simulation to evaluate the skills of holistic care of ICU nurse. Meanwhile, this study outlines the development of a standardized, four sites, curriculum-mapped training program using nurse-led team simulation in holistic nursing. Finally, this study assesses the feasibility and benefits of this educational intervention for nurses working and training in ICU.

## Methods

A standardized ICU simulation for holistic care was developed by an interdisciplinary working party comprising educationalists and healthcare professionals (from medical, nursing and respiratory therapy backgrounds). The aims of the course were to enhance holistic nursing focus on biological, psychosocial ad spiritual aspects including history taking, physical examination, communication and teamwork ( Crouch, 2016; McEvoy, 2008; Selimen, 2011).

The holistic nursing-focused courses were divided into evaluating stage, training stage and post-course assessment stage at four sites (respiratory ICU, pediatric ICU, newborn ICU and gynecological ICU). All training sessions were performed from January 2017 to October 2018.

### Different stages of this SBR program

**- At evaluating stage,** each course commenced with an introduction to simulation and a brief description of the core elements of holistic nursing. The short debriefing sessions (30 min) at evaluating stage was undergone in group to provide attendees opportunity to interact with facilitators. The skills of holistic nursing of team were evaluated by facilitators according to their performance in simulation. Senior nurses were trained as facilitators for rating team performance [“excellent”; “appropriate”; “fair”; “poor”] on four aspects of holistic nursing skills [history taking, physical examination, communication and teamwork] at the evaluating and training (at the second simulation) stages.

After initial evaluation at four sites, with the coordination of core members of education committee, the site-specific simulation scenario was developed according to the deficiency found at evaluating stage ( Table 1).

**Table 1.** Curriculum-mapped topics with standardized scenarios and common elements for holistic nursing

Curriculum-mapped scenario in different sites	Simulation technique
Management of the acutely respiratory distress patient in respiratory ICU	High-fidelity SimMan 3G (with computer-controlled vital signs that allowed changes in patient characteristics to be simulated), life-size manikins, actor of family, actor of orderly, role-play and simulation exercise
Stabilization of the post-cardiac surgery patients in pediatrics ICU	High-fidelity SimBaby (with computer-controlled vital signs that allowed changes in patient characteristics to be simulated), life-size manikins, actor of family, actor of orderly, role-play and simulation exercise
Emergency resuscitation of the shock newborn in newborn ICU	High-fidelity SimNBAI (with computer-controlled vital signs that allowed changes in patient characteristics to be simulated), life-size manikins, actor of family, role-play and simulation exercise
Management of the postpartum hemorrhage in gynecological ICU	High-fidelity SimMom (with computer-controlled vital signs that allowed changes in patient characteristics to be simulated), life-size manikins, actor of family, role-play and simulation exercise

Standardized scenario outlines were given to course facilitators to provide educational consistency across sites. Finally, the course employed a variety of simulation techniques and incorporating key skills of holistic nursing ( Table 1).

Scenarios were piloted by several team members whose were considered critical and holistic care experts and refinements were made to establish content and face validity of the scenarios and skills. This case based simulation ensures that nurses have the opportunity to practice the skills necessary to evaluate and confront critical patient with holistic approaches.

**- At training stage**, Further, all the detail introductions of holistic nursing were introduced and mailed to all attendees before the training days. The introduction emphasizes that during holistic health assessment and care, communication involves feedback between the recipient and the transmitter of information for effective gathering of data. Free expression by the patient with little or no inhibition is important during holistic health assessment. Additionally, It is essential for the healthcare professional and the client to understand each other’s language.

Training sessions with twice simulations of one scenario by each team were held during prescheduled day where staffing and acuity allowed. In situ evaluation and training allowed nurses to familiarize themselves with the exact equipment that they would be expected to use in live situations on real patients. First simulation of each team was considered as preparation step for formal in-training assessment. Then, the assessment of team performance was undergone at the second simulation of ICU scenario. Then, in an unoccupied ICU room, all resuscitation equipment and devices, Siman 3G, Simon 3G, SimBaby or SimNBAI were set up in addition to the normal supplies in each ICU room.

Each team consisted of 4 nurses, 1 standardized family and 1 standardized physician. Randomly, attendees were responsible for history taking, physical examination, communication and teamwork in managing of ICU patient. Each simulated scenario lasted up to 20 min and was followed by a group debriefing session lasting ∼40 min that followed a debriefing model of description, analysis and application as previously described ( Jaye, 2015; Yang 2017). The debriefing faculty comprised facilitators and educationalists, both experienced in simulation training ( Beaubien, 2003), and they facilitated courses on four sites to achieve consistency of scenarios and learning objectives, as in previous standardized multicentre simulation programs ( Birns, 2014).

**- At post-course workplace assessment stages:** At the post-course workplace assessment stages, these pre-trained senior nurses are responsible for evaluation the attendee’s application frequency [“always”; “often”; “occasionally”; “rarely”] of learnt skills on real-world practice.

Attendee’s self-assessment at post-course stage

-.Self-assessment of confidence [“very confident of”; “somewhat confident of”; “confident of”; “not confident of”] of the holistic nursing skills

### Ethics statement

This study was approved by the Ethics Committee of Taipei Veteran General Hospital (No. 2016010BC) and complies with the principles of the Declaration of Helsinski Guidelines. In agreement with these standards, informed consent was obtained from each participant.

## Results/Analysis

In general, attendees commented that simulation was a valuable training modality addressing holistic nursing rarely taught formally in cross-disciplinary scenario.

### Participants

Basically, the distribution of mean age of nursing attendees, percentage of ICU nurse with more than 1-year clinical experience, percentage of having previous experience of holistic care training, and percentage of have last holistic care training within 6 months were not different among four sites ( Table 2).

**Table 2.** Characteristics of nursing attendees at four sites

Characteristic	respiratory ICU (n=16)	pediatrics ICU (n=16)	newborn ICU (n=16)	gynecological ICU (n=16)
Mean age (years)	38±5	37±7	33±11	34±9
Percentage of nurses with more than 1-year clinical experience (%)	50% (n=8)	56% (n=9)	38% (n=6)	43% (n=7)
Percentage of having previous experience of holistic care training (%)	62% (n=10)	44% (n=7)	38% (n=6)	50% (n=8)
Pre-intervention duration of last training to day of entering current study in those having previous holistic care training (%)				
Before 6 month	40% (4/10)	57%(4/7)	50%(3/6)	50%(4/8)
Within 6 month	60% (6/10)	43% (3/7)	50% (3/6)	50% (4/8)

### The evaluation improve the performance of team in holistic nursing across groups

In comparison with the performance at evaluating stage, the general performance of teams was significantly improved at the training stage. Notably, lower percentage of team were graded as “poor” in performance of holistic care than their corresponding performance at evaluating stage ( Table 3).

**Table 3.** Holistic care performance of teams (at each site, four team with 16 nurses, n=4 in each team) of nursing attendees at four sites at evaluating and training stages

at evaluating stage	Team’ holistic nursing performance for holistic care of ICU scenario within time limit			
	respiratory ICU	pediatrics ICU	newborn ICU	gynecological ICU
Distribution of team-performance that graded as *“excellent/appropriate/fair/poor”*	*0/1/2/1*	*0/1/2/1*	*0/1/1/2*	*1/0/1/2*
-Percentage of graded as “ *poor*” for team-performance	*25%*	*25%*	*50%*	*50%*
**at training stage**	**Team’ holistic nursing performance at the second simulation of ICU scenario**			
	*respiratory ICU*	pediatrics ICU	newborn ICU	gynecological ICU
Distribution of team-performance that graded as “ *excellent/appropriate/fair/poor*” for holistic care	*2/0/2/0*	*2/1/1/0*	*2/1/1/0*	*1/2/0/1*
-Percentage of graded as “ *poor*” for team-performance	*0%*	*0%*	*0%*	*25%*

In ICU scenarios, facilitators globally graded the team-performance as “excellent” [complete >75% (>3/4) of expected skills], “appropriate”[complete 50-75% (2-3/4) of expected skills], “fair” [complete 25-50% (1-2/4) of expected skills]. “poor” [complete <25% (<1/4) of expected skills].

### The increased application of holistic care is associated with more confident of attendees on practicing holistic care

Table 4 revealed that, at the post-course stage, the acceptable frequency of clinical application of holistic care observed by bedside nursing facilitators were associated with good confidence of nursing attendees about clinical practice of holistic care.

**Table 4.** Workplace evaluation by bedside senior nurses and self-assessment at post-course stage

Distribution of frequency	senior nurses workplace assessments of clinical application of holistic nursing skills			
	respiratory ICU	pediatrics ICU	newborn ICU	gynecological ICU
*[“* *always* */* *often*/ *occasionally*/ *rarely* *”]*	19/17/29/35%	18/33/19/30%	22/20/24/34%	30/31/11/28%
*-“* *always* *+* *often+* *occasionally*”(%)	65%	70%	66%	72%
frequency of application are grades as “ **always**” [75-100% of times], “ **often**” [50-75% of times], “ **occasionally**” [ 25-50% of times], “ **rarely**” [<25% of times]				
*Distribution of confidence*	**self-assessment confidence of nursing attendees for the clinical application of holistic nursing skills**			
	***respiratory ICU***	**pediatrics ICU**	**newborn ICU**	**gynecological** **ICU**
[“ *very* *confident of*”, “ *somewhath* *confident of*”, “ *not* *confident of*”, “ *very not* *confident of*”]	22/30/34/14%	31/29/22/16%	25/29/32/14%	35/25/28/12%
*“* *very* *confident of* + *somewhat* *confident of*”(%)	52%	60%	54%	60%

## Discussion

Nurses are described as the eyes and ears of the ICU because their holistic approaching skills enable timely medical intervention to achieve the best outcome of patients ( Jacobs, 2007; Meyer, 2005; Harris, 2002). However, there is literature to suggest that nurses are not always able to provide the holistic care they had been taught in the classroom ( Crouch, 2016; King, 2006; Henderson, 2002; McEvoy, 2008). In view of the great influence of holistic care on treatment and more effective nursing, the health-care systems in many countries in recent decades have tried to promote holistic care by applying changes to the educational systems ( Selimen, 2011; Zamanzadeh, 2015). Nonetheless, recent studies show that concepts of holistic care still need to be enhanced in critical care ( Bullington, 2013; Gravel, 2012).

Simulation-based medical education (SBME) with deliberate practice (DP) is superior to traditional clinical medical education in achieving specific clinical skill ( Khan, 2011; McGaghie, 2011). Simulation-based research (SBR) studies use pre-post designs, usually involving an initial observation of the phenomenon of interest performed in a simulated environment, followed by the introduction of an intervention (training, new procedure, new equipment, etc) and repeated measures taken in simulated environment ( Portela, 2015). A particular strength of SBR is that it enables evaluation of specific skills without putting real patients (or trainees) at risk. Notably, our pilot study is a combination of SBME and SBR to segment holistic care of ICU nurses.

In holistic care, very good history taking skills are essential for the gathering of the general health history ( Clements, 2015). This encompasses the biographical data and subjective data on the patient’s physical, psychosocial, and spiritual state, which involves the use of a combination of verbal and nonverbal skills. In order to get more detailed information, objective data are required, which encompasses a physical examination. Keeping accurate and legible records after history taking and physical examination enhances care planning, implementation and evaluation, and also continuity of care ( Crouch, 2016; Briggs, 2017; Adams, 2017). Taken together, history taking, physical examination, effective communication and teamwork are the crucial elements for the success of holistic care.

In Miller’s framework for assessing clinical competence, the lowest level of the pyramid is knowledge (knows), followed by competence (knows how), performance (shows how), and action (does) ( Miller, 1990). In our study, we assessed the knowledge, competence and performance of nursing attendees at evaluation and training stages. Additionally, the action level was assessed at post-course stage by workplace observation by bedside senior nurses. Particularly, in our study, “action” focuses on what occurs in practice rather than what happens in a simulation. Especially, workplace-based methods of assessment target this highest level of the pyramid and collect information about attendees’ performance in their everyday practice.

Simulation is designed to place participants in complex and often uncomfortable clinical situations, so that they may experience provision of clinical care in a protected environment. The experience is then discussed and reflected upon in a supportive environment with knowledgeable clinical faculty ( Gaba, 2001; Perkins, 2007).

In addition to its benefits, the potential harms need to avoid during simulation. In our study, the performances of attendees are evaluated by direct observation rather than video recording to protect the privacy of attendees. Challenging clinical topics were purposefully incorporated into scenarios to stimulate a facilitated discussion around clinical protocols and good practice. Accordingly, the feedback skills of our senior facilitators are continuously trained in our institution to avoid the psychological harm of standardized patient actors or individuals managing the simulation ( Gaba, 2013; Yang, 2017). Overall, we provide a safe environment, both physically and psychologically, a good debriefing, avoiding death scenarios with early learners, and providing follow-up after simulation or even mentioning the possibility of patient death during pre-briefing to above aforementioned harms to participants ( Gaba, 2013; Calhoun, 2013).

Simulation is a useful method of facilitating experiential learning in holistic care and was shown to be feasible as part of a multiple sites program ( Gaba, 2001). Holistic care-specific course affords the educational opportunity to use a full range of simulation modalities; high-fidelity, low-fidelity, part-task trainers, patient actors and role-play exercises. So, post-course self-assessment showed improvement in attendees’ confidence in holistic care skills as part of a multidisciplinary team.

The strengths of this study include efforts were taken to achieve standardization of course structure with prescriptive scenario outlines and cross-site faculty working in both course design and delivery. This learning model makes learning objectives relevant and useful. Standardization facilitates the format to be transferable to multiple simulation sites and to future courses.

Limitations of the study include the lack of use of a control arm to compare those that did not attend a simulation session. So, the positive effect of the analysis needs to be examined cautiously due to the lack of a control group. Workplace assessments create a self-directive learning environment that is essential for continuing professional development. It should be noted that the positive effects of the simulation training were limited by the nature of self-reporting confidence. Notably, self-efficacy rating (self-reporting confidence in our study) is based on the principle of motivation theory where individuals have a relatively good understanding of their own abilities and weaknesses to motivate their transference ( Bandura, 1997). So, it is reasonable to observe that an attendee’s rating confidence has a good correlation with real-world application assessed by senior nurses.

## Conclusion

This multi-site simulation-based research (SBR) demonstrating that simulation training for holistic care is a valuable method for providing experience in ICU nurses for skills of history taking, physical examination, communication and teamwork.

## Take Home Messages

• Hoistic nursing of intensive care unit (ICU) patients encompasses diverse chalenges in psychoogical, social, and spiritual cares requiring reguar evaluation, training and assessment
• This simuation-based research (SBR), through of evauating, training and post-course assessment stages, aim to standardize the ICU training of hoistic nursing
• Specificaly, the curriculum-mapped scenarios were developed according to the deficiency of each site after evaluating stage.
• Post-course workplace assessment by senior nurses revealed the high frequency of clinical application of holistic nursing by attendees; post-course self assessment revealed a high attendee’s confidence of holistic approaching in ICU.

## Notes On Contributors

Dr. Tsu-Hui Shiao, M.D., Attending physicians of Department of Chest Medicine, Taipei Veterans General Hospital, Taipei, Taiwan; Department of Medicine, National Yang-Ming University, Taipei, Taiwan.

Dr. Chen-Yi Wu, M.D., Attending physicians of Department of Dermatology, Taipei Veterans General Hospital, Taipei, Taiwan; Department of Medicine, National Yang-Ming University, Taipei, Taiwan.

Prof. Ying-Ying Yang, M.D., PhD, MPH, Director of Clinical Skills Center, Department of Medical Education, Taipei Veterans General Hospital, Taiwan; Department of Medicine, National Yang-Ming University, Taipei, Taiwan.

Prof. Shi-Chuan Chang, M.D., PhD, Head, Department of Chest Medicine, Taipei Veterans General Hospital, Taiwan; Professor, Institute of critical care medicine, National Yang Ming University, Taiwan, Department of Medicine, National Yang-Ming University, Taipei, Taiwan.

Prof. Boaz Shulruf, M.D., PhD, MPH, BSc, University of New South Wales Sydney Australia (UNSW), Associate Professor of; Medical Education Research Chair: Admissions and Re-enrolment Committee Coordinator: PhD Research in Medical Education School Student Ethics Officer (SSEO) of UNSW.

Prof. Ling-Yu Yang, M.D., PhD, Chief of Department of Medical Education, Taipei Veterans General Hospital, Taiwan; Department of Medicine, National Yang-Ming University, Taipei, Taiwan.

Prof. Chen-Huan Chen, M.D., Dean, National Yang-Ming University, School of Medicine, Taiwan.

Prof. Fa-Yauh Lee, M.D., Deputy Superintendent of Taipei Veterans General Hospital, Taiwan.

## Declarations

The author has declared that there are no conflicts of interest.

## Ethics Statement

This study was approved by the Ethics Committee of Taipei Veteran General Hospital (No. 2016010BC) and complies with the principles of the Declaration of Helsinki Guidelines.

## External Funding

The work was supported by the grants (MOST-106-2511-S-010-001-MY3) of Ministry of Health and Welfare, Taiwan Association of Medical Education, National Yang-Ming University (107F-M01-0603) and Taipei Veteran General Hospital (V106-EA-007).
