# Supplementary material for: Pilot simulation-based research for evaluation, training and assessment of holistic nursing at four intensive care unit sites
Source: MedEdPublish (2016). 2019 Mar 7;8:40. [Version 1] doi: 10.15694/mep.2019.000040.1 (PMC10712602; doi:10.15694/mep.2019.000040.1)
Supplement: Peer review history

# Reviewer report: Reviewer response for version 1

**DOI:** 10.21956/mep.19766.r29680

**Published:** 2019-06-30

**Authors:** Ken Masters

**Affiliations:** 1 Sultan Qaboos University

This review has been migrated. The reviewer awarded 3 stars out of 5 The paper deals with a Pilot study on simulation-based research for evaluation, training and assessment of holistic nursing at four intensive care unit sites. The authors clearly lay out the problem of needing nurses to be able to respond appropriately and holistically in ICU settings, and the need for adequate training. A course with simulators was designed, and a pilot of the course was run to test the feasibility and success of the course. The course with its pre and post work appears to have been well designed, although more details are required (see comments below).Some areas needing attention:• At the end of the methods, there is a description of the evaluation process that is not well described at all. I don’t understand if these are part of an evaluation form, or observation, etc. This needs to be described in more detail. This is really important, because the effect of the data presented is pivotal to the understanding the success of the course, but the process of evaluation has to be clearly understood. • To assist in understanding the point above, if there is an evaluation form, it would be useful if the form(s) could be supplied in the Appendix or supplementary files.• There is too little information given about the course. The authors should remember that one of the most valuable aspects of such a paper is lessons for others to replicate. For this to exist, it would be useful if the course outline and at least two of the scenarios could be given.• The conclusion is really too short, and under-sells the findings in the paper. The authors need to elaborate on this.• Finally, the paper suffers from a large number of language errors that are sometimes just irritating, but sometimes interfere with understanding. The authors should proof-read their paper far more carefully before submitting.So, the course and the study itself appears to have value for the reader, but the authors have undermined their efforts in the presentation of the paper. I believe that, if the authors were to adequately address these points in a revised version of the paper, I would be prepared to increase my rating.

Reviewer Expertise:

NA

No decision status is available

# Reviewer report: Reviewer response for version 1

**DOI:** 10.21956/mep.19766.r29682

**Published:** 2019-03-09

**Authors:** Leila Niemi-Murola

**Affiliations:** 1 University of Helsinki

This review has been migrated. The reviewer awarded 4 stars out of 5 Thak you for the opportunity to review this interesting manuscript. Intensive Care Unit is a challenging working environment and it is not an easy task to learn how to work there. Thus, simulation is an ideal introduction method for new staff. It was not quite clear for the reader if the learning sessions were full-scale simulations, but that became apparent when the authors reported the devices used in the study. One of the intended learning outcomes of the simulations was history taking. Perhaps the authors mean communication with the patient, because the patient’s history has been written in the patient’s files, written in the emergency room. The ICU patients are often very ill, many of them intubated, and verbal communication is a challenge. In pediatric and neonatal ICUs, holistic care also involves communication with the patient’s parents.The results are very encouraging and I am sure that the authors will continue with this simulation-based mastery learning.

Reviewer Expertise:

NA

No decision status is available

# Reviewer report: Reviewer response for version 1

**DOI:** 10.21956/mep.19766.r29681

**Published:** 2019-03-07

**Authors:** Felix Silwimba

**Affiliations:** 1 University of Lusaka

This review has been migrated. The reviewer awarded 5 stars out of 5 this is good study that brings out issues of nursing practice in critical areas. the study clearly demonstrated the need for regular evaluation of practice for quality improvement and assurance. it has re-emphasized the importance of experiential learning and the role of millers pyramid in competence acquisition by nurses.

Reviewer Expertise:

NA

No decision status is available
